# Adaptive fractional fuzzy sliding mode control of microgyroscope based on backstepping design

**DOI:** 10.1371/journal.pone.0218425

**Published:** 2019-06-24

**Authors:** Xiao Liang, Juntao Fei

**Affiliations:** College of IoT Engineering, Hohai University, Changzhou, China; National University of Singapore, SINGAPORE

## Abstract

In this paper, a robust sliding mode control (SMC) based on backstepping technique is studied for a microgyroscope in the presence of unknown model uncertainties and external disturbances using adaptive fuzzy compensator and fractional calculus. At first, the dynamic of microgyroscope is transformed into analogically cascade system to guarantee the application of backstepping design. Then a novel fractional differential sliding surface is proposed which integrates the capacities of the fractional calculus and SMC. In order to reduce the chattering in SMC, a fuzzy logical system is utilized to approximate the external disturbances. In addition, fractional order adaptive laws are derived to estimate the damping and stiffness coefficients and angular velocity online based on Lyapunov stability theory which also guarantees the stability of the closed loop system. Finally, simulation results signify the robustness and effectiveness of the proposed control schemes and the comparison of root mean square error under different fractional orders and integer order are given to demonstrate the better performance of proposed controller.

## Introduction

Microgyroscope has many applications in military and civil fields such as navigation, automobile and traffic etc. due to their superior features in angular velocity measurement. However, constrained by manufacturing process and design principle, it is difficult to meet desired requirements and its performance is sensitive to time varying system parameters, external disturbances, ambient conditions including temperature and pressure and so on. In order to obtain better dynamic performance, lots of robust control methods have been applied to microgyroscope for many years. Park[1] proposed an adaptive control scheme with velocity estimation to compensate fabrication imperfects so as to operate insusceptibly in varying environments for a z-axis microgyroscope. In [2], two adaptive controllers were developed to tune the natural frequency of the drive axis for a vibrational microgyroscope. Adaptive neural sliding mode control algorithms were proposed for the unknown system dynamics and nonlinearities in the microgyroscope in [3–4]. A direct model reference adaptive control scheme with an estimating observer to modify disturbance was investigated which ensured the resonant oscillations of the microgyroscope in [5]. The tuning algorithm for systems parameters is derived based on Lyapunov stability theorem which guarantees the stability of the closed–loop system. By constructing suitable Lyapunov functions and combing with matrix inequality technique, new simple sufficient conditions are presented for stochastic delayed cellular neural networks in [6–7] and global asymptotic stability of the cohen-grossberg neural network models in [8–9] respectively.

Fractional calculus which expends the order of differential and integral from integer to fraction has been studied for three centuries. In recent years, more and more attention has been paid on its application in controller design instead of a pure theoretical mathematical subject owing to its higher modeling accuracy and degree of freedom compared to integer order controllers. Some researches about fractional calculus have been studied in [10–13]. Fractional order controllers were employed for microgrid in [14]. A fractional model was established to solve some physical problem in [15–16]. A model reference adaptive control strategy with fractional operators was demonstrated to improve the plant dynamics in [17]. A local fractional differential equation of fractal dimensional order is applied to a non-differentiable model of the LC-electric circuit in [18]. Some researches about the solvability for nonlinear fractional differential equations have been studied in [19–21].

The sliding mode control (SMC) technique is considered to be an effective control scheme for robust control which has been applied to both linear and nonlinear systems. The main idea of SMC is to choose a linear manifold of the state variables such as deviations and their derivatives as sliding surface and then design a control law for driving and constraining the system state into the previous designed sliding surface. SMC has shown great superiorities in dealing with nonlinear systems with uncertainties which is benefit from its robustness and insensitivity to parameters variation and external disturbances. Sling mode control and observations were focused on in [22] for complex industrial systems because of the advantages above. An adaptive novel SMC using neural network and fuzzy system are designed for the uncertain nonlinear system in [23] [24]. An adaptive SMC with a new adaptive law, whose adaptive gains was inversely proportional to the sliding variables, offered the fast dynamic and reduced chattering for robot manipulators in [25]. Neural network and fuzzy system are also utilized to deal with uncertainties and suppress the harmonics for active power filter in [26] [27] [28] [29].

SMC applies not only to integer order systems, but also to fractional order systems. Thus fractional order calculus can also be incorporated in sliding mode control [30–31]. Chen et al. [30] proposed an adaptive sliding mode control scheme for a fractional order nonlinear system with uncertainties. A fractional order fuzzy sliding mode controller was designed for robotic manipulators which retained the advantages of SMC and reduced the chattering simultaneously in [31].

Backstepping method has been well known for its recursive and systematic design in nonlinear feedback systems [32] [33] [34]. The concept of backstepping control is to choose appropriate functions of the state variables as virtual controls for subsystems and then design control laws based on Lyapunov functions. A simplified adaptive backstepping scheme was proposed for a full-car active suspension system with external disturbances in [35]. An adaptive backstepping controller was proposed for vehicle active suspensions in [36] to guarantee the stability of the attitude of vehicle and the improvement of ride comfort. Unfortunately, backstepping control scheme does not work well for systems with discontinuous disturbance and parameter variations. So it is usually combined with other intelligent control methods such as sliding mode control, fuzzy control and so on. An adaptive sliding mode controller based on backstepping technique was proposed for robotic manipulator in [37] which estimates the system uncertainties and external disturbances by the adaptive laws. Park et al. [38] designed a backstepping integral sliding mode controller based on T-S fuzzy model for an Interior Permanent Magnet Synchronous Motor. A backstepping fractional order sliding mode control was developed for power systems and microgyroscope system respectively which showed good dynamic performances and great robustness compared to traditional methods in [39–40]. Feng et. al [41] proposed a novel adaptive Super-Twisting sliding mode control for a microgyroscope.

In this paper, in order to incorporate the advantages of fractional control, sliding mode control, fuzzy control and backstepping control, an adaptive fractional fuzzy sliding mode controller based on backstepping design is proposed for a microgyroscope. The output trajectory of microgyroscope track the reference trajectory accurately and effectively and the estimation of system parameters have been verified to converge to their true values asymptotically. The main contributions of this paper are emphasized as follows:

1. The superior characteristic of this designed controller is that a fractional order term is adopted in the sliding manifold which generates an extra degree of freedom and makes the design of control law more flexible, consequently the performance of the closed loop system has been improved a lot compared to the traditional SMC whose sliding surface is based on integer order calculus of the state variables.
2. Based on backstepping fractional sliding mode control scheme, a fuzzy logical system is designed to deal with the unknown uncertainties and external disturbances which weakened the chattering phenomenon. Furthermore, adaptive algorithm for parameters of microgyroscope is derived based on Lyapunov stability theory, which guarantees the stability of the closed-loop system and the unknown parameters of microgyroscope system can be identified on line simultaneously. In general, the method proposed in this paper both improves the system performance and enhancing system robustness against model uncertainties and external disturbances as well.

This paper is organized as follows: In section 2, the dynamics of microgyroscope is described. The structure of backstepping fractional sliding mode control and adaptive fractional fuzzy sliding mode control based on backstepping technique are proposed in section 3 and section 4 respectively. Simulation results are shown in section 5 and finally for the conclusions.

## Materials and methods

In this section, the mathematical model of z-axis microgyroscope is described, and the preliminary of fractional calculus is introduced, then for solving the trajectory tracking problem of microgyroscope system with unknown model uncertainties and external disturbances, an adaptive fractional fuzzy sliding mode controller based on backstepping design is proposed based on Lyapunov theory.

### Dynamics of microgyroscope

**Fig 1:**
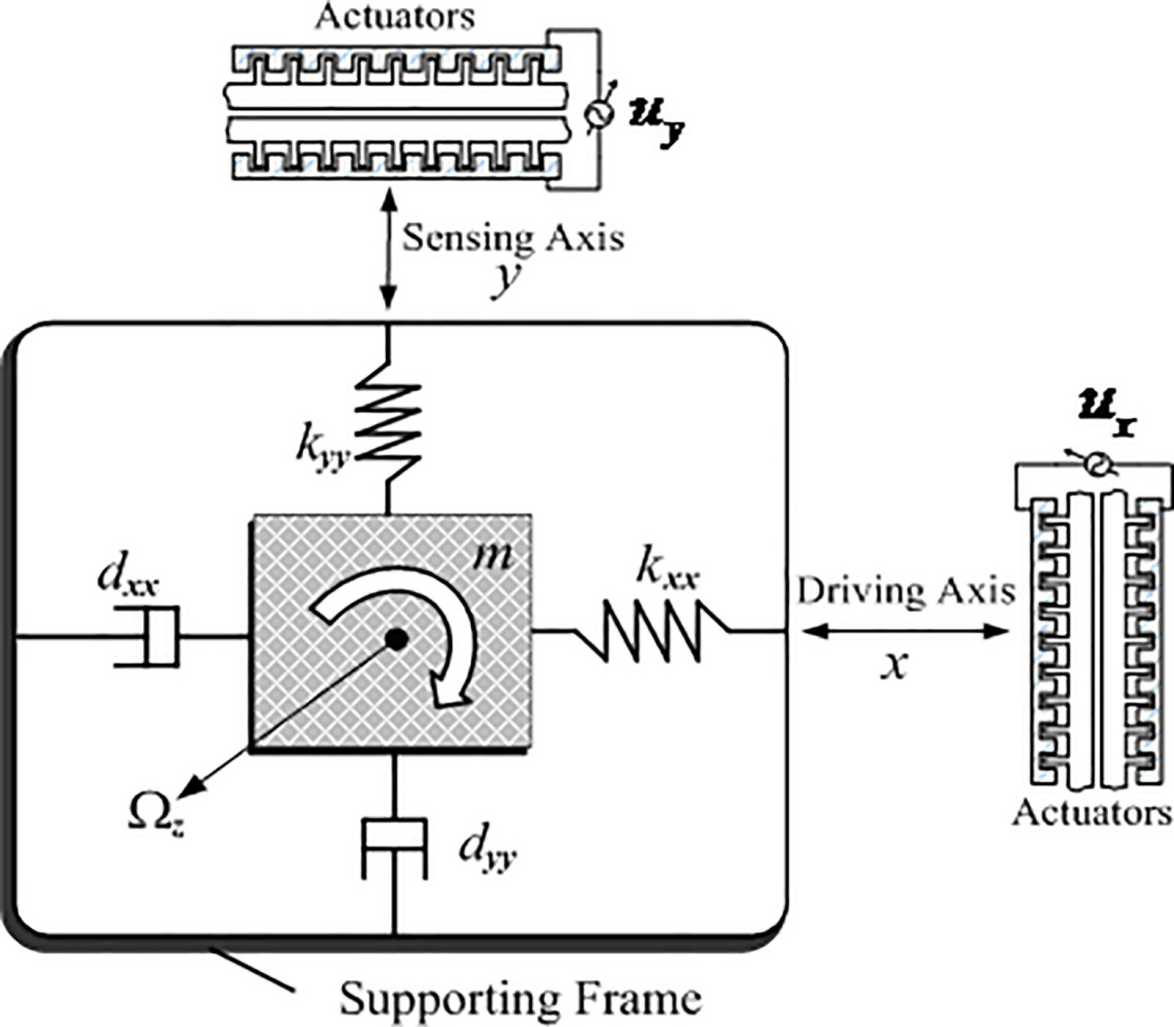
Schematic diagram of a z-axis microgyroscope.

**Fig 2:**
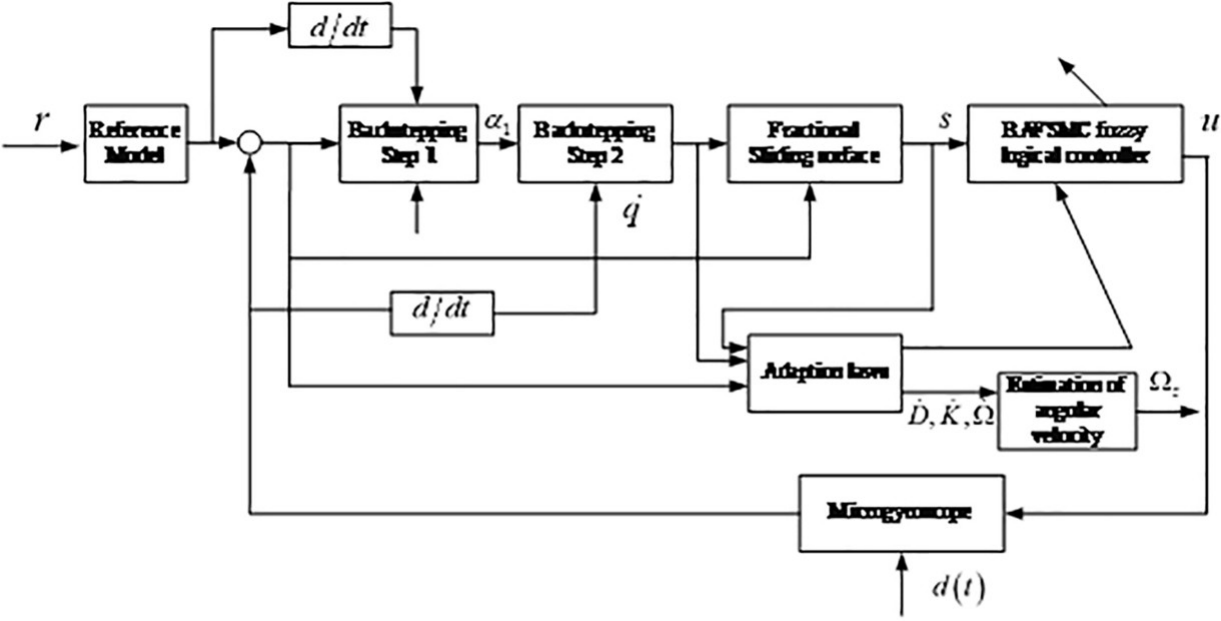
The architecture of the adaptive backstepping fractional fuzzy sliding mode controller.

The microgyroscope is composed of a proof mass, sensing mechanisms and electrostatic actuation used to force an oscillatory motion and velocity of the proof mass and to sense the position. In order to achieve the dynamics of the MEMS, some assumptions have been made: 1) the motion of the proof mass is limited to x and y axis as shown in Fig 1; 2) the microgyroscope rotates at a constant angular velocity; 3) the centrifugal forces is neglected. Under the above assumptions, the dynamics of the microgyroscope can be simplified as follows:

$$\begin{array}{l} m\ddot{x}+d_{x}\dot{x}+[k_{x}-m(\Omega_{y}{}^{2}+\Omega_{z}{}^{2})]x+m\Omega_{x}\Omega_{y}y=u_{x}+2m\Omega_{z}\dot{y}\\ m\ddot{y}+d_{y}\dot{y}+[k_{y}-m(\Omega_{x}{}^{2}+\Omega_{z}{}^{2})]y+m\Omega_{x}\Omega_{y}x=u_{y}-2m\Omega_{z}\dot{x} \end{array}$$ (1)

where *m* is the mass of proof mass, *d*_*x*,*y*_ and *k*_*x*,*y*_ are damping and spring coefficients terms along x- and y-axis respectively. Ω_*x*,*y*,*z*_ are the angular velocity along each axis, and *u*_*x*,*y*_ are the control forces in *x* and *y* directions.

Considering fabrication defects, which may cause extra coupling between x- and y- axis, the dynamics for a z-axis microgyroscope is revised as:

$$\begin{array}{l} m\ddot{x}+d_{xx}\dot{x}+d_{xy}\dot{y}+k_{xx}x+k_{xy}y=u_{x}+2m\Omega_{z}\dot{y}\\ m\ddot{y}+d_{xy}\dot{x}+d_{yy}\dot{y}+k_{xy}x+k_{yy}y=u_{y}-2m\Omega_{z}\dot{x} \end{array}$$ (2)

In the above equations, *d*_*xx*_ and *d*_*yy*_ are damping terms; *k*_*xx*_ and *k*_*yy*_ are spring coefficients terms; *d*_*xy*_ and *k*_*xy*_ are coupled damping and spring terms, respectively.

Dividing both sides of Eq (2) by proof mass *m*, reference length *q*_0_ and natural resonance frequency *ω*_0_ simultaneously results:

$$\begin{array}{l} \ddot{x}+d_{xx}\dot{x}+d_{xy}\dot{y}+\omega_{x}{}^{2}x+\omega_{xy}y=u_{x}+2\Omega_{z}\dot{y}\\ \ddot{y}+d_{xy}\dot{x}+d_{yy}\dot{y}+\omega_{xy}x+\omega_{y}{}^{2}y=u_{y}-2\Omega_{z}\dot{x} \end{array}$$ (3)

which is the nondimensional dynamics of microgyroscope.

In (3),

$$\begin{array}{c} \frac{d_{xx}}{m\omega_{0}}\to d_{xx},\frac{d_{xy}}{m\omega_{0}}\to d_{xy},\frac{d_{yy}}{m\omega_{0}}\to d_{yy}\\ \frac{k_{xx}}{m\omega_{0}{}^{2}}\to \omega_{x}^{2},\frac{k_{xy}}{m\omega_{0}{}^{2}}\to \omega_{xy},\frac{k_{yy}}{m\omega_{0}{}^{2}}\to \omega_{y}^{2},\frac{\Omega_{z}}{m\omega_{0}}\to \Omega_{z} \end{array}$$ (4)

Through the equivalent transformation, the vector form of the model is described as:

$$\ddot{q}+D\dot{q}+Kq=u-2\Omega \dot{q}$$ (5)

where

$$q=\left[\begin{array}{c} x\\ y \end{array}\right],D=\left[\begin{array}{cc} d_{xx} & d_{xy}\\ d_{xy} & d_{yy} \end{array}\right],K=\left[\begin{array}{cc} \omega_{x}^{2} & \omega_{xy}\\ \omega_{xy} & \omega_{y}^{2} \end{array}\right],u=\left[\begin{array}{c} u_{x}\\ u_{y} \end{array}\right],\Omega =\left[\begin{array}{cc} 0 & -\Omega_{z}\\ \Omega_{z} & 0 \end{array}\right]$$ (6)

### Backstepping fractional sliding mode control

#### Preliminary introduction of fractional order

As the extended form of differentiation and integration, Caputo(C), Riemann-Liouville(RL), and Grunwald-Letnikov(GL) definitions are the three most commonly used definitions in engineering, science and economics fields, especially the Caputo fractional order calculus which happens to be adopted in this paper.

The Caputo fractional derivative of order *α* of function *f*(*x*) is denoted as:

$${}_{a}D{{}_{t}}^{\alpha }f\left(t\right)=\frac{1}{\Gamma \left(n-\alpha \right)}\int_{a}^{t}\frac{f^{\left(n\right)}\left(\tau \right)}{{\left(t-\tau \right)}^{\alpha -n+1}}d\tau ,\;n-1<\alpha <n$$ (7)

where *t* and *a* are the upper and lower bounds of the operator respectively and Γ is the Gamma function which satisfies:

$$\Gamma \left(\gamma \right)=\int_{0}^{\infty }e^{-t}t^{\gamma -1}dt$$ (8)

For convenience, _*a*_*D*_*t*_^*α*^ is replaced by *D*^*α*^ in the following parts.

It is noted that if *α* = 0, then the operation *D*^0^*f*(*x*) satisfies *D*^0^*f*(*x*) = *f*(*x*).

Fractional differential sliding mode surface is proposed in this part since its higher control precision compared to the integer order for the adjustable fractional order *α*. Backstepping control is usually applied to a class of special nonlinear dynamical systems which can be built from subsystems by choosing appropriate Lyapunov functions. Thanks to the recursive procedure, good tracking performance and global stability are guaranteed.

#### Design of backstepping fractional sliding mode control

Considering the system parameter variations and external disturbances, the dynamic of the MEMS gyroscope is described as follows:

$$\ddot{q}+(D+2\Omega )\dot{q}+Kq=u+d$$ (9)

where *d* denotes the lumped bounded uncertainties and disturbances which satisfies ‖*d*‖≤*ρ*, and *ρ* is a positive constant, referring to the upper bound of the uncertainties and disturbances.

For the application of backstepping technique, coordinate transformation of the dynamic is necessary.

Define two variables *x*_1_ and *x*_2_. Let

$$x_{1}=q,\;x_{2}=\dot{q}$$ (10)

Then a mathematical model of MEMS gyroscope can be expressed as follows:

$$\{\begin{array}{c} {\dot{x}}_{1}=x_{2}\\ {\dot{x}}_{2}=-\left(D+2\Omega \right)x_{2}-Kx_{1}+u+d \end{array}$$ (11)

Making the position vector *q* follow its desired trajectory strictly is the main object of the controller design. The specific controller design is divided into two steps.

Step 1: Assume that *q*_*r*_ is the ideal tracking value, then the tracking error vector can be defined as:

$$e_{1}=x_{1}-q_{r}$$ (12)

Then its time derivative is

$${\dot{e}}_{1}={\dot{x}}_{1}-{\dot{q}}_{r}=x_{2}-{\dot{q}}_{r}$$ (13)

Define the virtual control variable as:

$$\alpha_{1}=-c_{1}e_{1}+{\dot{q}}_{r}$$ (14)

where *c*_1_ is a constant and *c*_1_>0.

Define the new error variable as:

$$e_{2}=x_{2}-\alpha_{1}$$ (15)

Select a Lyapunov function as Eq (16):

$$V_{1}=\frac{1}{2}e_{1}{}^{T}e_{1}$$ (16)

By deriving both sides of (16) one can obtain:

$$\begin{array}{l} {\dot{V}}_{1}=e_{1}{}^{T}{\dot{e}}_{1}=e_{1}{}^{T}\left(x_{2}-{\dot{q}}_{r}\right)\\ \;=e_{1}{}^{T}\left(e_{2}-c_{1}e_{1}\right)\\ \;=e_{1}{}^{T}e_{2}-c_{1}e_{1}{}^{T}e_{1} \end{array}$$ (17)

If *e*_2_ = 0, then

$${\dot{V}}_{1}=-c_{1}e_{1}{}^{T}e_{1}\leq 0$$ (18)

Step 2: The time derivative of (15) is

$$\begin{array}{l} {\dot{e}}_{2}={\dot{x}}_{2}-{\dot{\alpha }}_{1}\\ =-\left(D+2\Omega \right)x_{2}-Kx_{1}+u+d-{\dot{\alpha }}_{1} \end{array}$$ (19)

A fractional order sliding mode surface is defined as:

$$s=\lambda_{1}e_{1}+\lambda_{2}D^{\alpha -1}e_{1}+\lambda_{3}e_{2}$$ (20)

where *λ*_1_,*λ*_2_,*λ*_3_ refer to the positive sliding surface parameters and *α*−1 is the fractional order of fractional derivate operation.

Taking the time derivative of *s*, we get:

$$\dot{s}=\lambda_{1}{\dot{e}}_{1}+\lambda_{2}D^{\alpha }e_{1}+\lambda_{3}{\dot{e}}_{2}$$ (21)

A new Lyapunov function is described as:

$$V_{2}=V_{1}+\frac{1}{2}s^{T}s$$ (22)

By making derivative of (22), we have:

$$\begin{array}{l} {\dot{V}}_{2}={\dot{V}}_{1}+s^{T}\dot{s}\\ =e_{1}{}^{T}e_{2}-c_{1}e_{1}{}^{T}e_{1}+s^{T}\left(\lambda_{1}{\dot{e}}_{1}+\lambda_{2}D^{\alpha }e_{1}+\lambda_{3}{\dot{e}}_{2}\right) \end{array}$$ (23)

where

$$e_{2}=\frac{s-\lambda_{1}e_{1}-\lambda_{2}D^{\alpha -1}e_{1}}{\lambda_{3}}$$ (24)

Then, Eq (24) is added into Eq (23), which yields

$$\begin{array}{l} {\dot{V}}_{2}=e_{1}{}^{T}e_{2}-c_{1}e_{1}{}^{T}e_{1}+s^{T}\left(\lambda_{1}{\dot{e}}_{1}+\lambda_{2}D^{\alpha }e_{1}+\lambda_{3}{\dot{e}}_{2}\right)\\ =-c_{1}e_{1}{}^{T}e_{1}+e_{1}{}^{T}\frac{s-\lambda_{1}e_{1}-\lambda_{2}D^{\alpha -1}e_{1}}{\lambda_{3}}+s^{T}\left[\lambda_{1}{\dot{e}}_{1}+\lambda_{2}D^{\alpha }e_{1}+\lambda_{3}\left(f+u+d-{\dot{\alpha }}_{1}\right)\right] \end{array}$$ (25)

where $f=-\left(D+2\Omega \right)\dot{q}-Kq=-\left(D+2\Omega \right)x_{2}-Kx_{1}$.

In order to keep ${\dot{V}}_{2}\leq 0$, the corresponding control law is designed as:

$$\begin{array}{l} u=-f-\rho \frac{s}{\left\|s\right\|}+{\dot{\alpha }}_{1}+\frac{1}{\lambda_{3}}\left(-\lambda_{1}{\dot{e}}_{1}-\lambda_{2}D^{\alpha }e_{1}-\frac{e_{1}}{\lambda_{3}}+\frac{\lambda_{2}se_{1}{}^{T}}{{\left\|s\right\|}^{2}\lambda_{3}}D^{\alpha -1}e_{1}\right)\\ =\left(D+2\Omega \right)\left(e_{2}+\alpha_{1}\right)+K\left(e_{1}+q_{r}\right)-\rho \frac{s}{\left\|s\right\|}+{\dot{\alpha }}_{1}\\ +\frac{1}{\lambda_{3}}\left(-\lambda_{1}{\dot{e}}_{1}-\lambda_{2}D^{\alpha }e_{1}-\frac{e_{1}}{\lambda_{3}}+\frac{\lambda_{2}se_{1}{}^{T}}{{\left\|s\right\|}^{2}\lambda_{3}}D^{\alpha -1}e_{1}\right) \end{array}$$ (26)

Since *s*^*T*^*e*_1_ = *e*_1_^*T*^*s*, substituting Eq (26) into Eq (25) results:

$$\begin{array}{l} {\dot{V}}_{2}=-c_{1}e_{1}{}^{T}e_{1}-\frac{\lambda_{1}}{\lambda_{3}}e_{1}{}^{T}e_{1}+s^{T}\lambda_{3}\left(d-\rho \frac{s}{\left\|s\right\|}\right)\\ \leq -c_{1}e_{1}{}^{T}e_{1}-\frac{\lambda_{1}}{\lambda_{3}}e_{1}{}^{T}e_{1}+\lambda_{3}\left(\left\|s\right\|\left\|d\right\|-\rho \left\|s\right\|\right)\\ \leq -c_{1}e_{1}{}^{T}e_{1}-\frac{\lambda_{1}}{\lambda_{3}}e_{1}{}^{T}e_{1}\leq 0 \end{array}$$ (27)

The derivative of *V*_2_ keeps negative semi- definite. According to Barbalart lemma, it can be proved $\underset{t\to \infty }{\lim}e_{1}\left(t\right)=0$, $\underset{t\to \infty }{\lim}s\left(t\right)=0$, that is to say the proposed control strategy can ensure the asymptotical stability of the closed loop system.

#### Adaptive fractional fuzzy sliding mode control based on backstepping technique

In previous controller design, the control law (26) is derived under the condition of the available parameter variations *D*,*K*,Ω and external disturbances *ρ*. On the contrary, these uncertainty bounds are unknown in actual systems. So for the better conduction of the backstepping fractional SMC system in practice, a good estimate of the unknown parameters with $\hat{D},\hat{K},\hat{\Omega }$ is necessary. Adaptive schemes combined are used online to collect data and adjust the parameters automatically. In addition, an adaptive fuzzy compensator $\hat{h}\left(s\right)$ is proposed to handle the chattering caused by the sliding mode surface. The architecture of the proposed adaptive fractional fuzzy sliding mode controller based on backstepping technique is shown in Fig 2.

Define the parameter estimation error as:

$$\begin{array}{l} \tilde{D}=\hat{D}-D\\ \tilde{K}=\hat{K}-K\\ \tilde{\Omega }=\hat{\Omega }-\Omega \\ {\tilde{\theta }}_{h}=\theta_{h}{}^{*}-\theta_{h} \end{array}$$ (28)

The adaptive control law *u*' can be derived as:

$$u^{'}=\left(\hat{D}+2\hat{\Omega }\right)\left(e_{2}+\alpha_{1}\right)+\hat{K}\left(e_{1}+q_{r}\right)+{\dot{\alpha }}_{1}-\hat{h}\left(s\right)+\frac{1}{\lambda_{3}}\left(-\lambda_{1}{\dot{e}}_{1}-\lambda_{2}D^{\alpha }e_{1}-\frac{e_{1}}{\lambda_{3}}+\frac{\lambda_{2}se_{1}{}^{T}}{{\left\|s\right\|}^{2}\lambda_{3}}D^{\alpha -1}e_{1}\right)$$ (29)

where

$$\hat{h}\left(s|\theta \right)={\left[{\hat{h}}_{1}\;{\hat{h}}_{2}\right]}^{T}={\left[\theta_{h1}{}^{T}\Phi \left(s_{1}\right)\;\theta_{h2}{}^{T}\Phi \left(s_{2}\right)\right]}^{T}$$ (30)

Assuming that $\hat{h}\left(s|\theta_{h}{}^{*}\right)=\rho \frac{s}{\left\|s\right\|}$, then the optimal parameters of fuzzy system is defined as:

$$\theta_{h}{}^{*}=\arg\underset{\theta_{h}\in \Omega_{h}}{\min}\underset{x\in R^{n}}{\left[\sup\left|\hat{h}\left(s|\theta_{h}\right)-\hat{h}\left(s|\theta_{h}{}^{*}\right)\right|\right]}$$ (31)

where Ω_*h*_ are the collections of parameter and *θ*_*h*_.

Substituting the control law (29) into $\dot{s}$ as in (21) results in:

$$\begin{array}{l} \dot{s}=\lambda_{1}{\dot{e}}_{1}+\lambda_{2}D^{\alpha }e_{1}+\lambda_{3}\left(f+u+d-{\dot{\alpha }}_{1}\right)\\ \;=\lambda_{3}\left(\left(\tilde{D}+2\tilde{\Omega }\right)\left(e_{2}+\alpha_{1}\right)+\tilde{K}\left(e_{1}+q_{r}\right)+d-\hat{h}\left(s\right)+\frac{1}{\lambda_{3}}\left(-\frac{e_{1}}{\lambda_{3}}+\frac{\lambda_{2}se_{1}{}^{T}}{\lambda_{3}{\left\|s\right\|}^{2}}D^{\alpha -1}e_{1}\right)\right) \end{array}$$ (32)

Define the Lyapunov function candidate as:

$$V=\frac{1}{2}e_{1}{}^{T}e_{1}+\frac{1}{2}s^{T}s+\frac{1}{2}tr\{\tilde{D}M^{-1}{\tilde{D}}^{T}\}+\frac{1}{2}tr\{\tilde{K}N^{-1}{\tilde{K}}^{T}\}+\frac{1}{2}tr\{\tilde{\Omega }P^{-1}{\tilde{\Omega }}^{T}\}+\frac{1}{2r}\sum_{i=1}^{2}{\tilde{\theta }}_{hi}{}^{T}{\tilde{\theta }}_{hi}$$ (33)

where *M* = *M*^*T*^>0, *N* = *N*^*T*^>0, *P* = *P*^*T*^>0 are positive definite matrices and *tr*{•} denotes the matrix trace operator.

Taking the time derivation on both sides of *V* yields

$$\begin{array}{l} \dot{V}=-c_{1}e_{1}{}^{T}e_{1}+e_{1}{}^{T}\frac{s-\lambda_{1}e_{1}-\lambda_{2}D^{\alpha -1}e_{1}}{\lambda_{3}}\\ +s^{T}\lambda_{3}\left(\left(\tilde{D}+2\tilde{\Omega }\right)\left(e_{2}+\alpha_{1}\right)+\tilde{K}\left(e_{1}+q_{r}\right)+d-\hat{h}\left(s\right)+\frac{1}{\lambda_{3}}\left(-\frac{e_{1}}{\lambda_{3}}+\frac{\lambda_{2}se_{1}{}^{T}}{{\left\|s\right\|}^{2}\lambda_{3}}D^{\alpha -1}e_{1}\right)\right)\\ +tr\{\tilde{D}M^{-1}{\dot{\tilde{D}}}^{{}^{T}}\}+tr\{\tilde{\Omega }P^{-1}{\dot{\tilde{\Omega }}}^{{}^{T}}\}+tr\{\tilde{K}N^{-1}{\dot{\tilde{K}}}^{{}^{T}}\}+\frac{1}{r}\sum_{i=1}^{2}{\tilde{\theta }}_{hi}{}^{T}{\dot{\tilde{\theta }}}_{hi}\\ =-c_{1}e_{1}{}^{T}e_{1}-\frac{\lambda_{1}e_{1}{}^{T}e_{1}}{\lambda_{3}}+s^{T}\lambda_{3}\tilde{D}\left(e_{2}+\alpha_{1}\right)+tr\{\tilde{D}M^{-1}{\dot{\tilde{D}}}^{{}^{T}}\}\\ +s^{T}\lambda_{3}\tilde{K}\left(e_{1}+q_{r}\right)+tr\{\tilde{K}N^{-1}{\dot{\tilde{K}}}^{{}^{T}}\}\\ +2s^{T}\lambda_{3}\tilde{\Omega }\left(e_{2}+\alpha_{1}\right)+tr\{\tilde{\Omega }P^{-1}{\dot{\tilde{\Omega }}}^{{}^{T}}\}\\ +s^{T}\lambda_{3}\left(\hat{h}\left(s|\theta^{*}\right)-\hat{h}\left(s\right)+d-\hat{h}\left(s|\theta^{*}\right)\right)+\frac{1}{r}\sum_{i=1}^{2}{\tilde{\theta }}_{hi}{}^{T}{\dot{\tilde{\theta }}}_{hi} \end{array}$$ (34)

Since *D* = *D*^*T*^,*K* = *K*^*T*^,Ω = −Ω^*T*^ and $s^{T}\tilde{D}\left(e_{2}+\alpha_{1}\right)={\left(e_{2}+\alpha_{1}\right)}^{T}\tilde{D}s$ are scalar, we have

$$\begin{array}{l} \lambda_{3}s^{T}\tilde{D}\left(e_{2}+\alpha_{1}\right)=\frac{1}{2}\left(\lambda_{3}s^{T}\tilde{D}\left(e_{2}+\alpha_{1}\right)+\lambda_{3}{\left(e_{2}+\alpha_{1}\right)}^{T}\tilde{D}s\right)\\ =tr\left\{\frac{1}{2}\lambda_{3}\left(\tilde{D}\left(e_{2}+\alpha_{1}\right)s^{T}+\tilde{D}s{\left(e_{2}+\alpha_{1}\right)}^{T}\right)\right\} \end{array}$$ (35)

Simultaneously, we obtained

$$\begin{array}{l} \lambda_{3}s^{T}\tilde{K}\left(e_{1}+q_{r}\right)=\frac{1}{2}\left(\lambda_{3}s^{T}\tilde{K}\left(e_{1}+q_{r}\right)+\lambda_{3}{\left(e_{1}+q_{r}\right)}^{T}\tilde{K}s\right)\\ =tr\{\frac{1}{2}\lambda_{3}\left(\tilde{K}\left(e_{1}+q_{r}\right)s^{T}+\tilde{K}s{\left(e_{1}+q_{r}\right)}^{T}\right)\}\\ 2\lambda_{3}s^{T}\tilde{\Omega }\left(e_{2}+\alpha_{1}\right)=\lambda_{3}s^{T}\tilde{\Omega }\left(e_{2}+\alpha_{1}\right)-\lambda_{3}{\left(e_{2}+\alpha_{1}\right)}^{T}\tilde{\Omega }s\\ =tr\{\lambda_{3}\left(\tilde{\Omega }\left(e_{2}+\alpha_{1}\right)s^{T}-\tilde{\Omega }s{\left(e_{2}+\alpha_{1}\right)}^{T}\right)\} \end{array}$$ (36)

Substituting (35) and (36) into (34) results:

$$\begin{array}{l} \dot{V}=-c_{1}e_{1}{}^{T}e_{1}-\frac{\lambda_{1}e_{1}{}^{T}e_{1}}{\lambda_{3}}+tr\{\tilde{D}\left(M^{-1}{\dot{\tilde{D}}}^{{}^{T}}+\frac{1}{2}\lambda_{3}\left(\left(e_{2}+\alpha_{1}\right)s^{T}+s{\left(e_{2}+\alpha_{1}\right)}^{T}\right)\right)\}\\ +tr\{\tilde{K}\left(N^{-1}{\dot{\tilde{K}}}^{{}^{T}}+\frac{1}{2}\lambda_{3}\left(\left(e_{1}+q_{r}\right)s^{T}+s{\left(e_{1}+q_{r}\right)}^{T}\right)\right)\}\\ +tr\{\tilde{\Omega }\left(P^{-1}{\dot{\tilde{\Omega }}}^{{}^{T}}+\lambda_{3}\left(\left(e_{2}+\alpha_{1}\right)s^{T}-s{\left(e_{2}+\alpha_{1}\right)}^{T}\right)\right)\}\\ +\frac{1}{r}\sum_{i=1}^{2}{\tilde{\theta }}_{hi}{}^{T}\left(r\lambda_{3}s_{i}\Phi \left(s_{i}\right)+{\dot{\tilde{\theta }}}_{hi}\right)+\lambda_{3}s^{T}\left(d-\hat{h}\left(s|\theta^{*}\right)\right) \end{array}$$ (37)

where *i* = 1,2 represents the two-axis vector.

In order to guarantee $\dot{V}\leq 0$, the online adapting laws for parameters are as follows:

$$\begin{array}{l} {\dot{\hat{D}}}^{T}={\dot{\tilde{D}}}^{T}=-\frac{1}{2}\lambda_{3}M\left(\left(e_{2}+\alpha_{1}\right)s^{T}+s{\left(e_{2}+\alpha_{1}\right)}^{T}\right)\\ {\dot{\hat{K}}}^{T}={\dot{\tilde{K}}}^{T}=-\frac{1}{2}\lambda_{3}N\left(\left(e_{1}+q_{r}\right)s^{T}+s{\left(e_{1}+q_{r}\right)}^{T}\right)\\ {\dot{\hat{\Omega }}}^{T}={\dot{\tilde{\Omega }}}^{T}=-\lambda_{3}P\left(\left(e_{2}+\alpha_{1}\right)s^{T}-s{\left(e_{2}+\alpha_{1}\right)}^{T}\right)\\ {\dot{\theta }}_{hi}=-{\dot{\tilde{\theta }}}_{hi}=r\lambda_{3}s_{i}\Phi \left(s_{i}\right),i=1,2 \end{array}$$ (38)

Substituting (38) into (37), it is obvious that

$$\begin{array}{l} \dot{V}=-c_{1}e_{1}{}^{T}e_{1}-\frac{\lambda_{1}e_{1}{}^{T}e_{1}}{\lambda_{3}}+\lambda_{3}s^{T}\left(d-\hat{h}\left(s|\theta^{*}\right)\right)\\ \leq -c_{1}e_{1}{}^{T}e_{1}-\frac{\lambda_{1}e_{1}{}^{T}e_{1}}{\lambda_{3}}+\lambda_{3}\left(\left\|s\right\|\left\|d\right\|-\rho \frac{s^{T}s}{\left\|s\right\|}\right)\\ \leq -c_{1}e_{1}{}^{T}e_{1}-\frac{\lambda_{1}e_{1}{}^{T}e_{1}}{\lambda_{3}}+\lambda_{3}\left(\left\|s\right\|\left\|d\right\|-\rho \left\|s\right\|\right)\\ \leq -c_{1}e_{1}{}^{T}e_{1}-\frac{\lambda_{1}e_{1}{}^{T}e_{1}}{\lambda_{3}}\leq 0 \end{array}$$ (39)

$\dot{V}$ is proved to be negative semi-definite which means $V,s,\tilde{D},\tilde{K},\tilde{\Omega }$ are all bounded. According to (32), $\dot{s}$ is also bounded. Integrating $\dot{V}$ with respect to time, we have $\int_{0}^{t}c_{1}e_{1}{}^{T}e_{1}+\frac{\lambda_{1}e_{1}{}^{T}e_{1}}{\lambda_{3}}+\lambda_{3}\left(\left\|s\right\|\left\|d\right\|-\rho \left\|s\right\|\right)\leq V\left(0\right)-V\left(t\right)dt$. Since *V*(0) is bounded and *V*(*t*) is bounded and non-increasing, it can be concluded that $\int_{0}^{t}c_{1}e_{1}{}^{T}e_{1}+\frac{\lambda_{1}e_{1}{}^{T}e_{1}}{\lambda_{3}}dt$ and $\int_{0}^{t}\lambda_{3}\left(\left\|s\right\|\left\|d\right\|-\rho \left\|s\right\|\right)dt$ are all bounded. According to Barbalart lemma, $\underset{t\to \infty }{\lim}e_{1}\left(t\right)=0$, $\underset{t\to \infty }{\lim}s\left(t\right)=0$, that is to say the tracking error and fractional sliding mode surface will asymptotically converge to zero which guarantees the stability of the gyroscope system.

## Results and discussions

A z-axis MEMS gyroscope dynamical model is chosen as a simulation example to validate the effectiveness of the proposed control strategy. The parameters of the microgyroscope are chosen as follows:

$$\begin{array}{l} m=1.8\times 10^{-7}kg,\;d_{xx}=1.8\times 10^{-6}\;N\;s/m,d_{yy}=1.8\times 10^{-6}\;N\;s/m,\\ d_{xy}=3.6\times 10^{-7}\;N\;s/m,\;k_{\mathrm{xx}}=63.955\;N/m,k_{yy}=95.92\;N/m,k_{xy}=12.779\;N/m. \end{array}$$

Assume that the unknown angular velocity is Ω_*z*_ = 100 rad/s. Then the non-dimensional gyroscope parameter matrices can be derived as follows:

$$D=\left[\begin{array}{cc} 0.01 & 0.002\\ 0.002 & 0.01 \end{array}\right],K=\left[\begin{array}{cc} 355.3 & 70.99\\ 70.99 & 532.9 \end{array}\right],\Omega =\left[\begin{array}{cc} 0 & -0.1\\ 0.1 & 0 \end{array}\right]$$ (40)

The membership functions of the fuzzy variable *s* are defined as:

$$\begin{array}{l} \mu_{NM}(s)=1/(1+\exp(5(s+3))),\mu_{ZO}(s)=\exp(-s^{2})\\ \mu_{PM}(s)=1/(1+\exp(5(s-3))) \end{array}$$ (41)

In this simulation example, reference trajectory is selected as *q*_*r*1_ = sin(4.17*t*), *q*_*r*2_ = 1.2sin(5.11*t*) and the initial states of the system are set as $q_{1}(0)=0.5,{\dot{q}}_{1}(0)=0,q_{2}(0)=0.5,{\dot{q}}_{2}(0)=0$. Choose the initial conditions of $\hat{D},\hat{K},\hat{\Omega }$ as ${\hat{D}}_{0}=0.95*D$, ${\hat{K}}_{0}=0.95*K$, ${\hat{\Omega }}_{0}=0.95*\Omega$. Select the sliding surface parameters *λ*_1_ = 55,*λ*_2_=10,*λ*_3_ = 1, the control parameters *c*_1_ = 200, *r* = 10000 and the adaptive gains *M* = *N* = *diag*(150,150),*P* = *diag*(20,20).

When the fractional order is set as *α* = 0.9 and the disturbance is applied as random signal *d* = [0.5*randn(1,1);0.5*randn(1,1)], the corresponding simulation results are shown in Figs 3–10.

**Fig 3:**
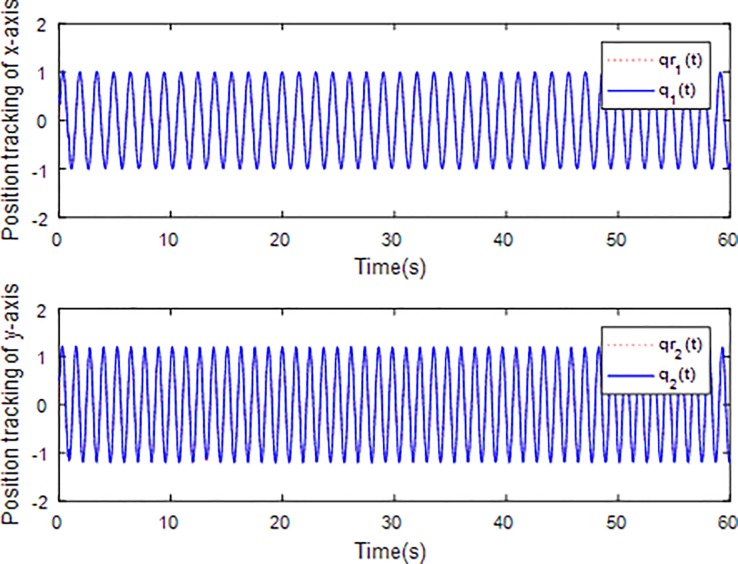
Tracking trajectory using fractional order sliding surface.

**Fig 4:**
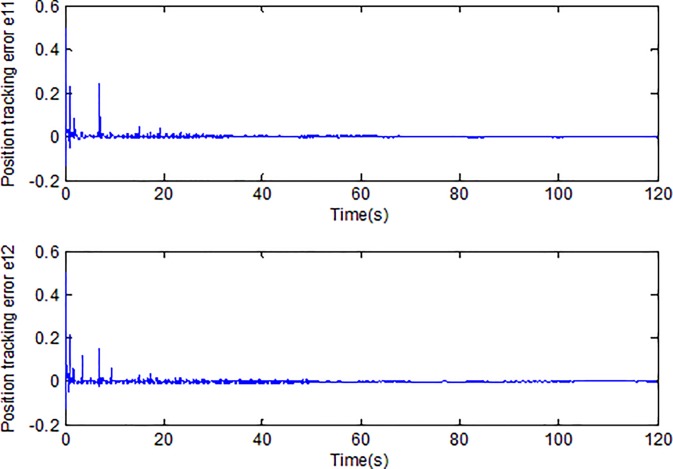
Tracking error using fractional order sliding surface.

**Fig 5:**
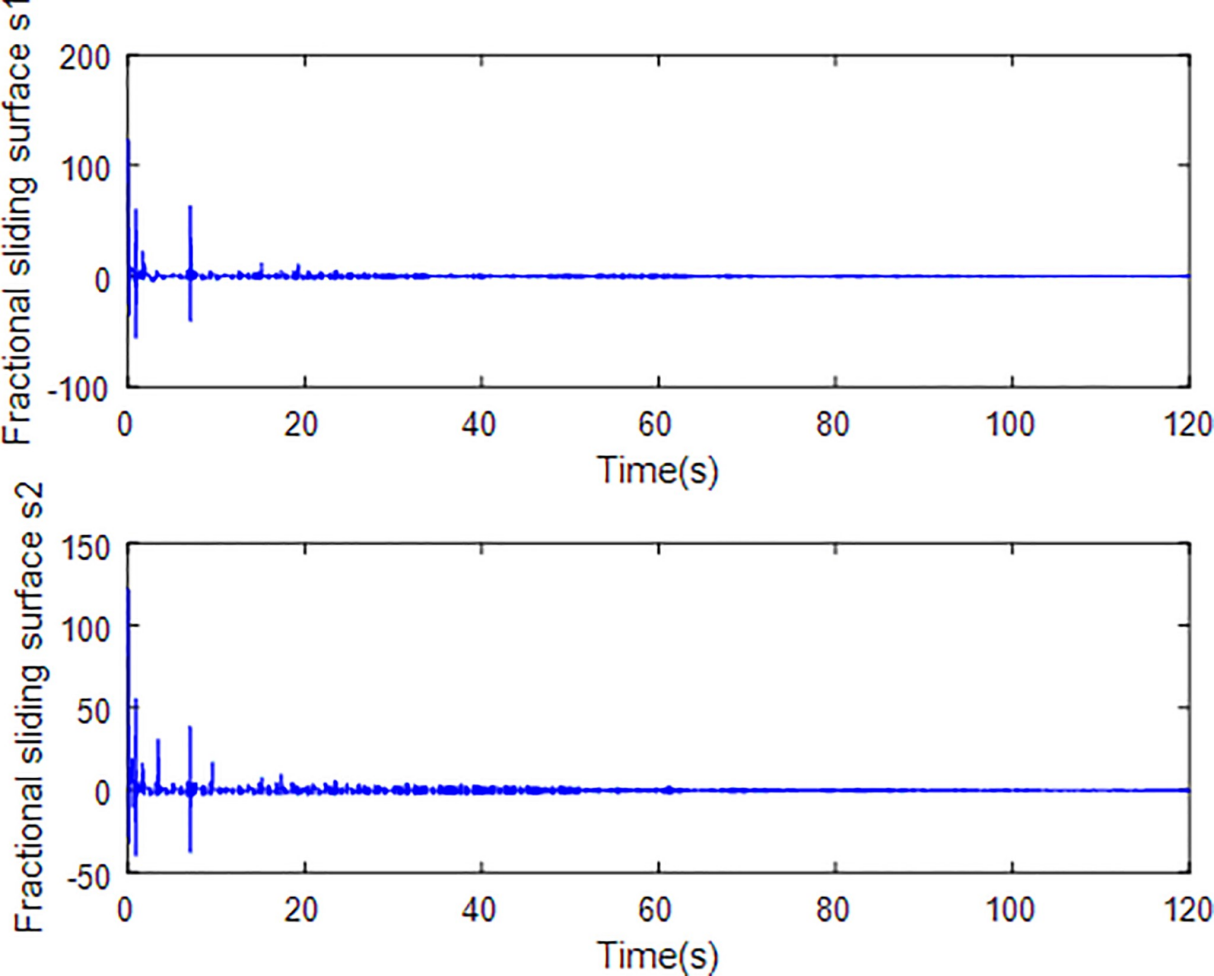
Fractional sliding surface.

**Fig 6:**
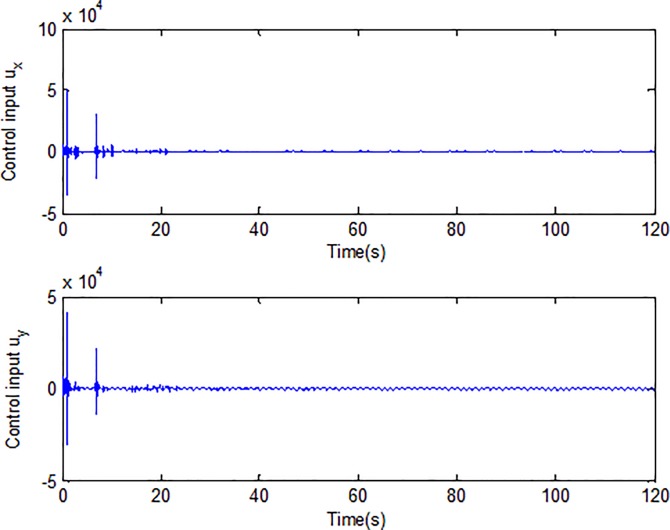
Control input signals.

**Fig 7:**
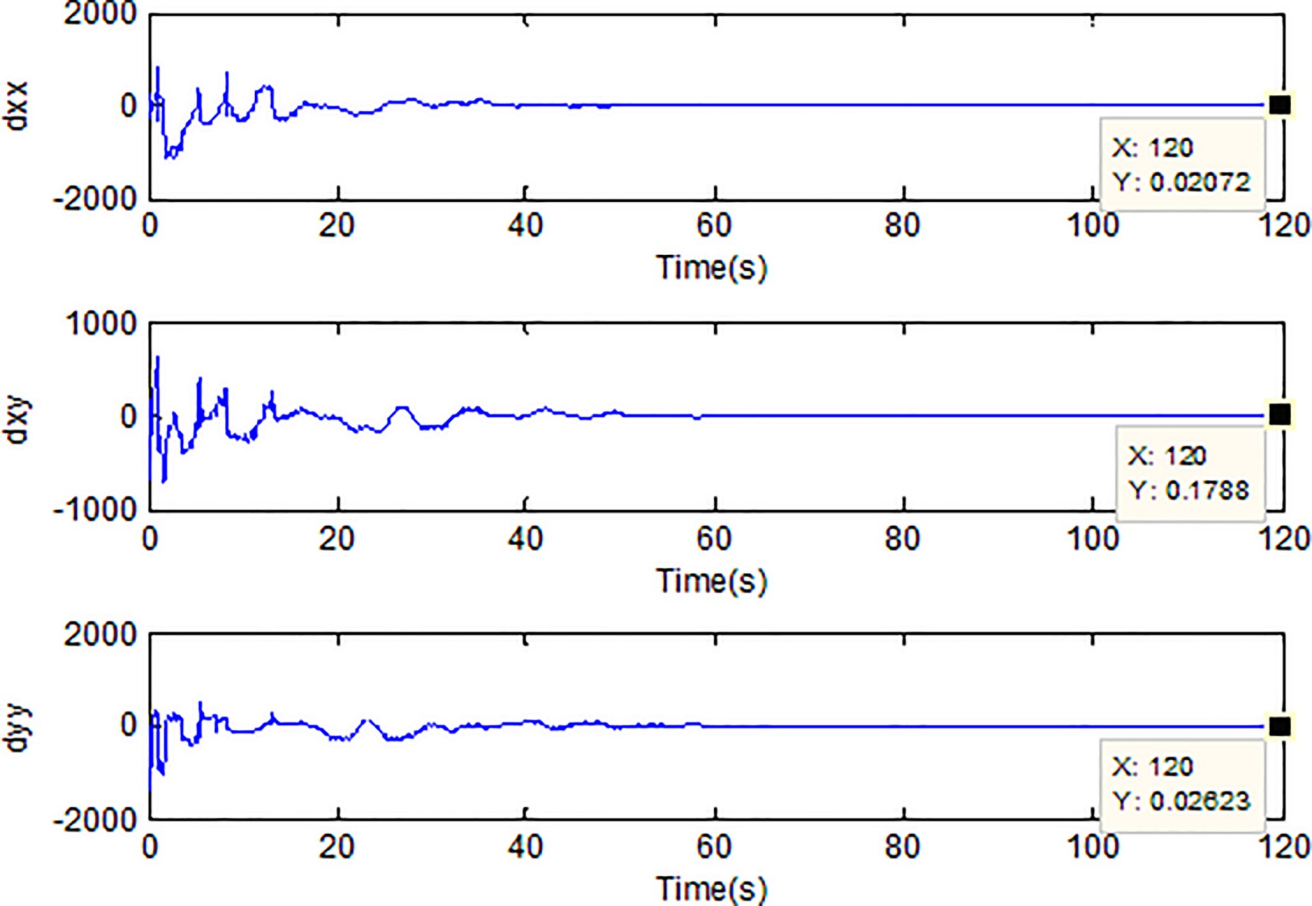
Adaption of damping coefficients of microgyroscope.

**Fig 8:**
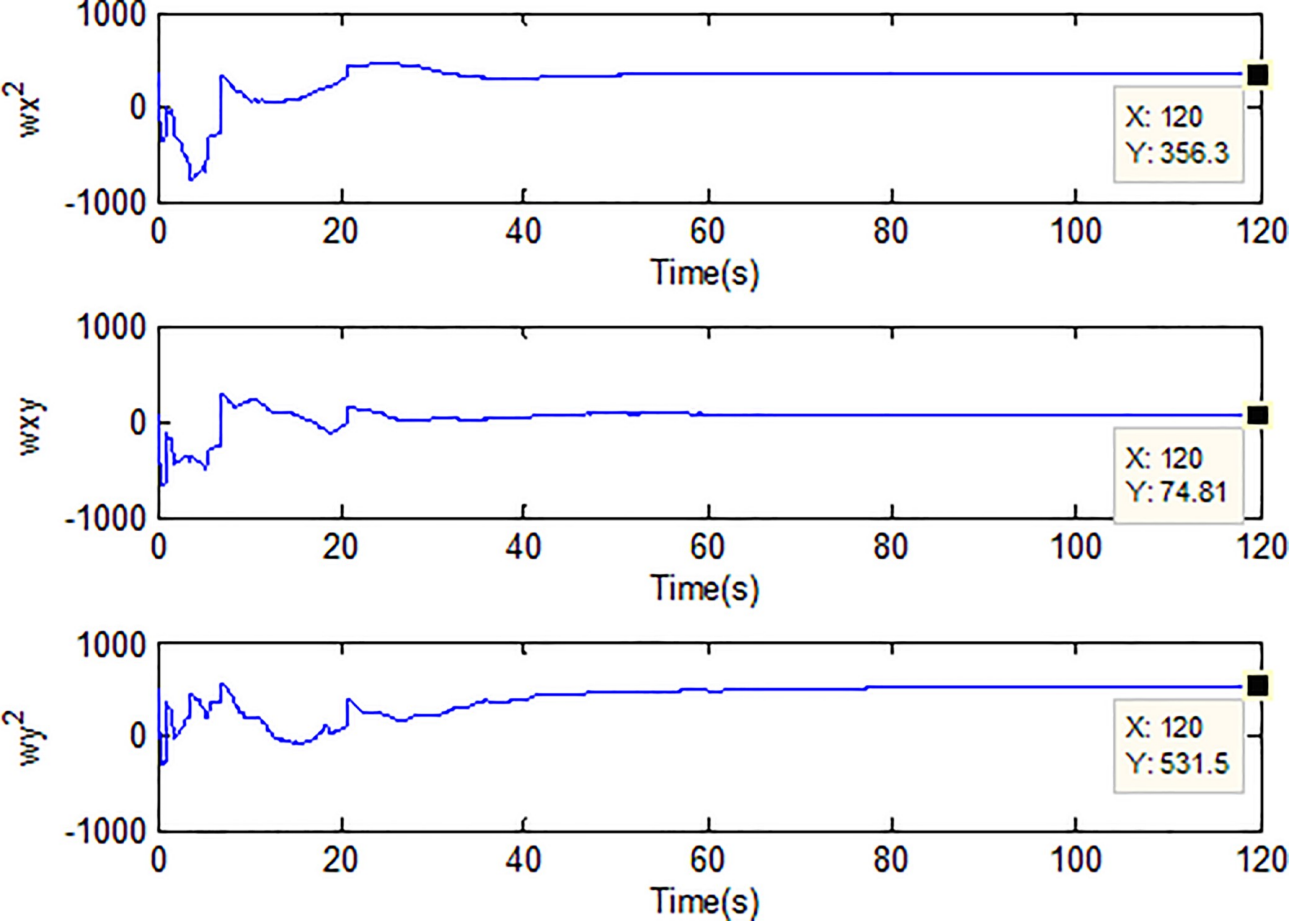
Adaption of spring constants of microgyroscope.

**Fig 9:**
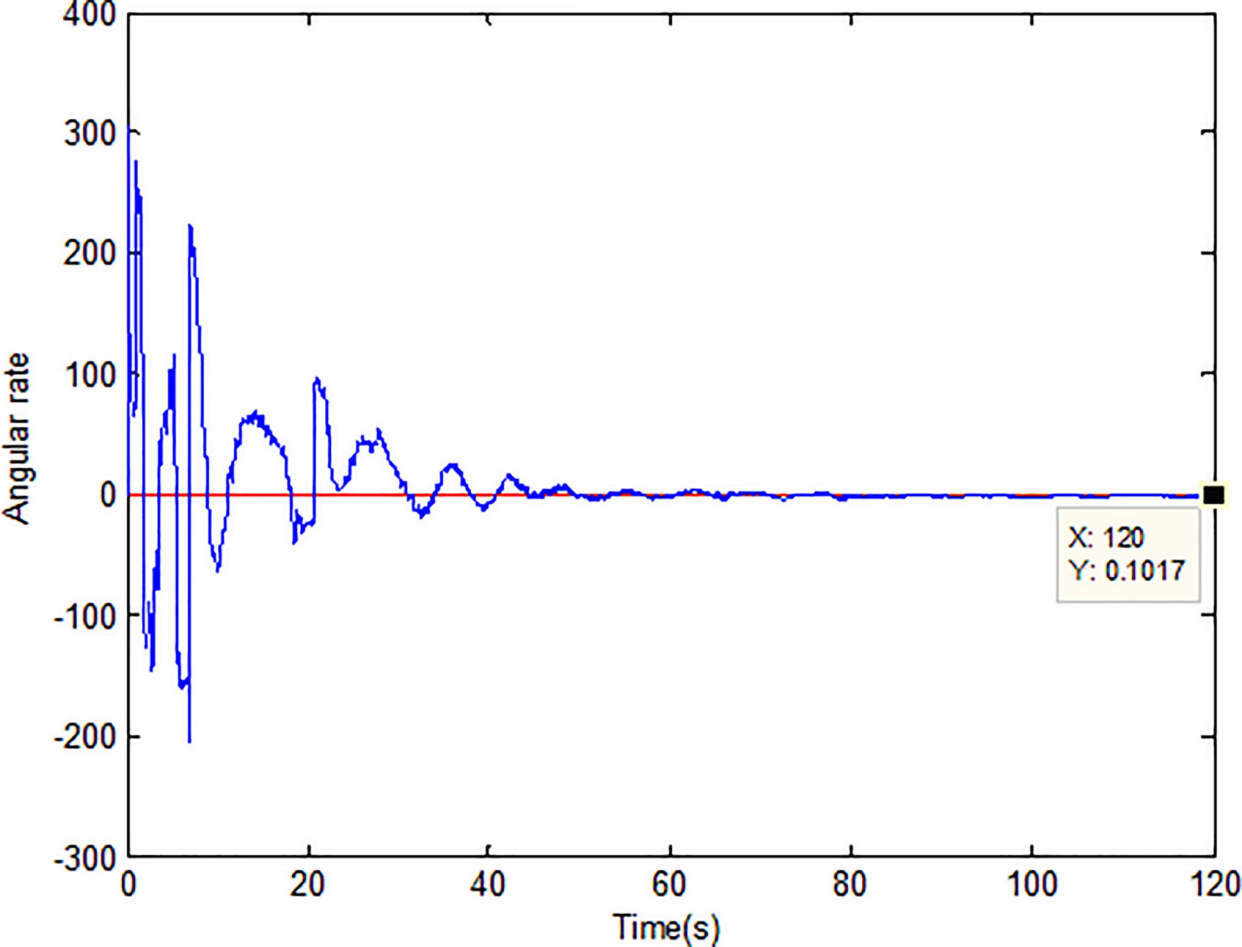
Adaption of angular velocity.

**Fig 10:**
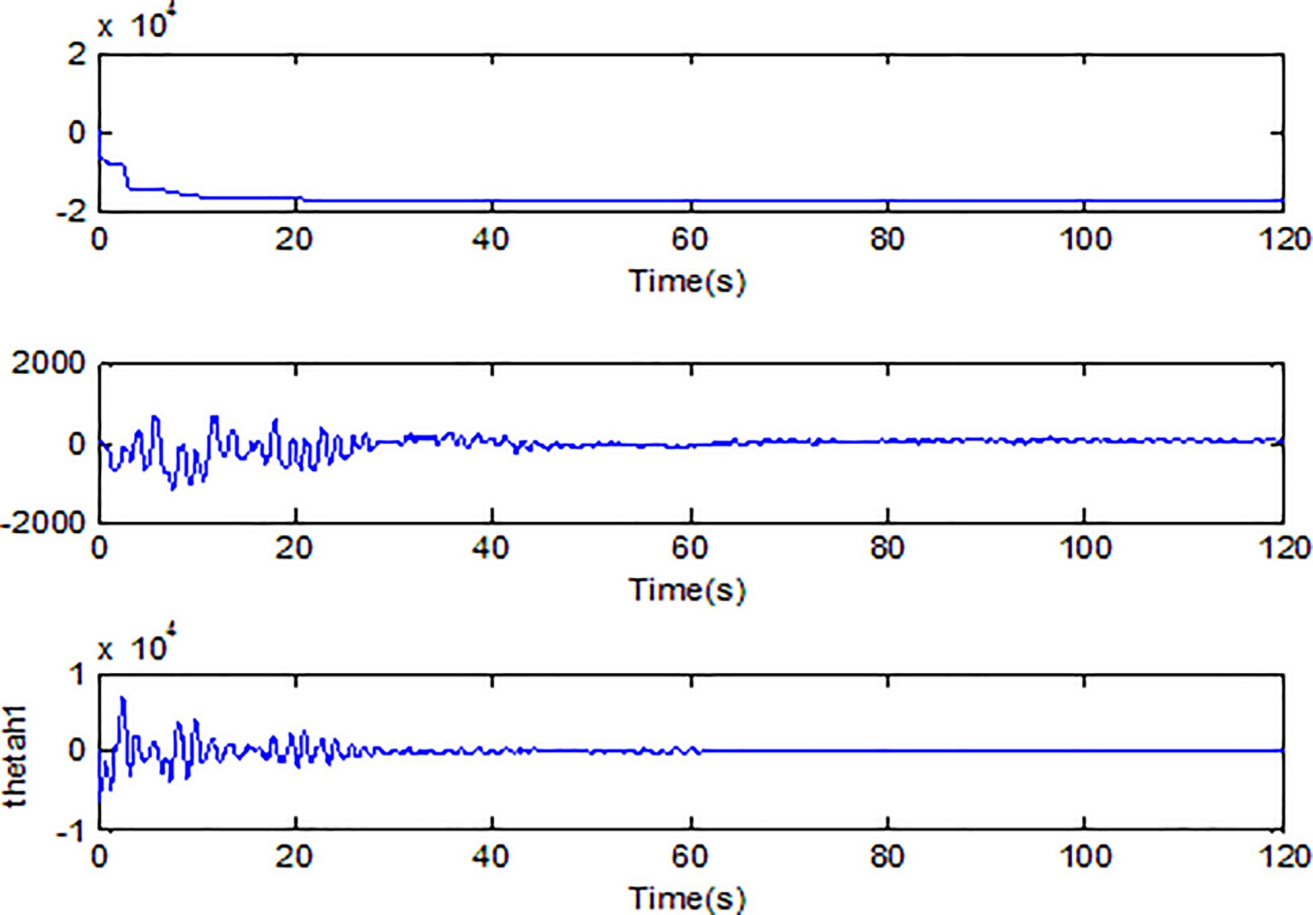
Adaption of *θ*_*h*_ along X axis.

Fig 3 describes the trajectories of the system states. It is obvious that the tracking performance is well achieved with the existence of external disturbance by the proposed adaptive fractional fuzzy sliding mode control based on backstepping technique. Fig 4 plots the tracking error of the microgyroscope system which converges to zero in a short time and guarantees the asymptotical stability of the system. In addition, the tracking error under the condition of *α* = 0.9 is demonstrated to be the lowest that will be introduced in detail below.

**Fig 11:**
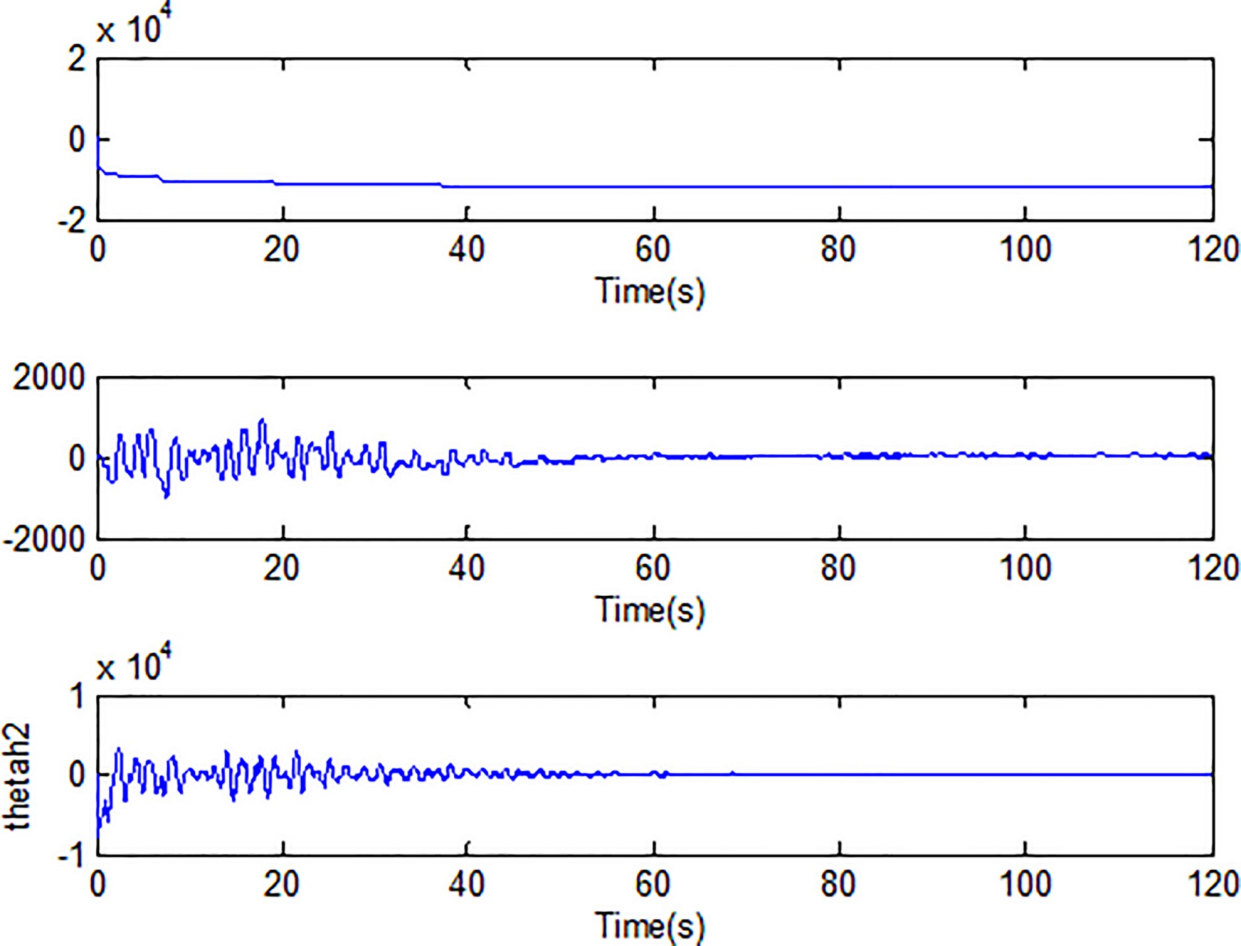
Adaption of *θ*_*h*_ along Y axis.

Fig 5 depicts the convergence of the fractional sliding surface *s*. It is intuitive that the sliding surface converges to zero within a short time which ensures that the trajectory of the system attains to sliding surface. In Fig 6, the time evolution of the input control signal is brought. The chattering is effectively reduced as a result of the approximation for switching function of fuzzy system. Fig 7 and Fig 8 draw the adaption of the system parameter matrix *D* and *K* respectively. With persistent sinusoidal signals, the estimation of *D* and *K* have been verified to converge to their true values which allows the existence of small range of errors. Fig 9 describes the adaption of angular velocity whose estimate also converges to its actual value. Fig 10 and Fig 11 depict the adaption of fuzzy parameter *θ*_*h*_ along X and Y axis respectively. It is obvious that the parameter reaches a steady state after 40 seconds.

**Fig 12:**
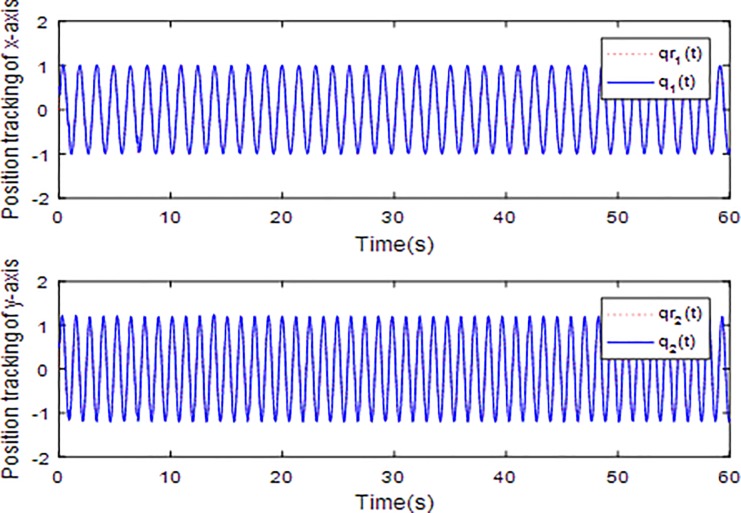
Tracking trajectory using integer order sliding surface.

**Fig 13:**
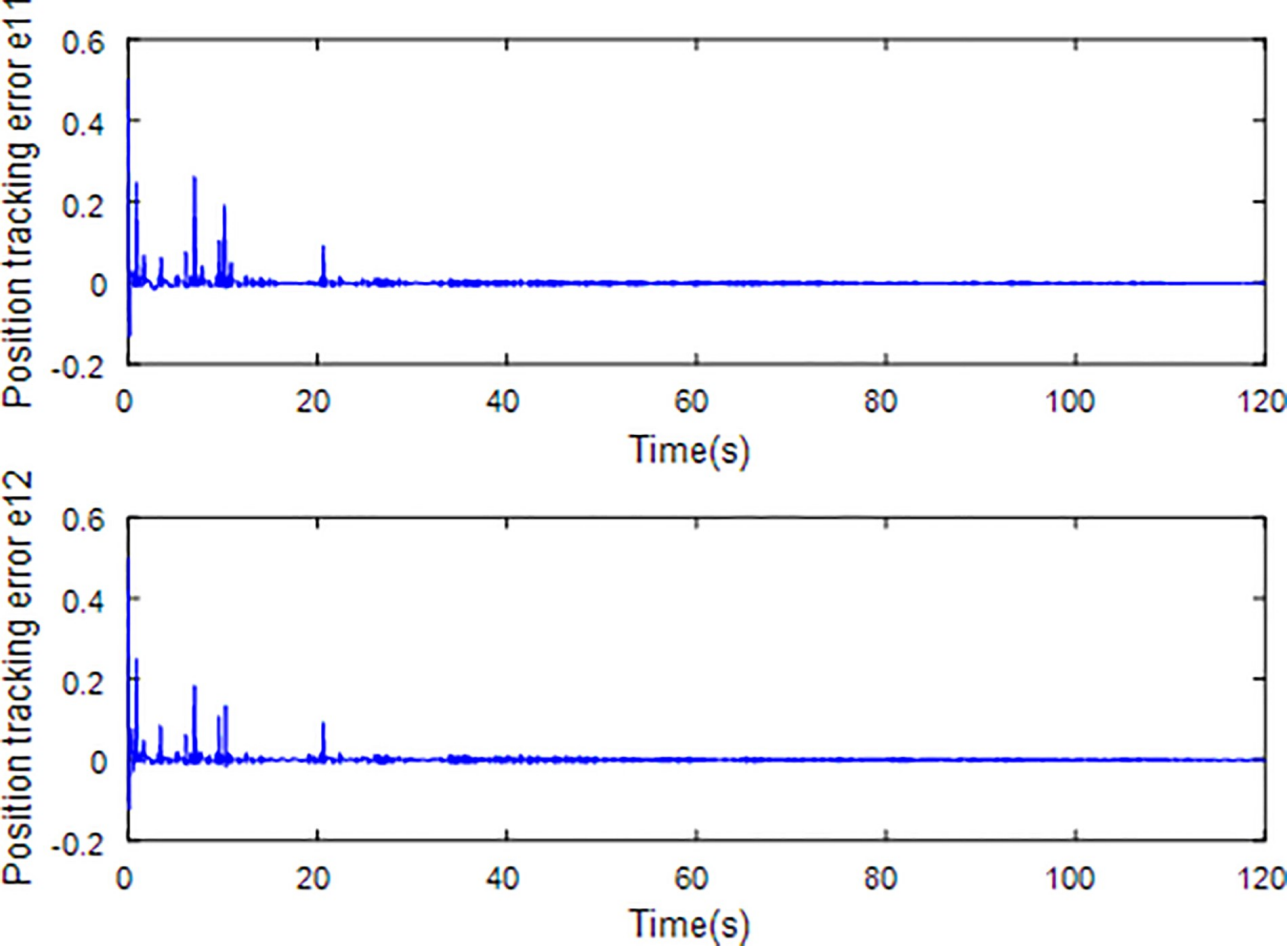
Tracking error using integer order sliding surface.

Fig 12 and Fig 13 plot the tracking trajectories and tracing errors of microgyroscope along x-axis and y-axis respectively using integer sliding mode controller. It can be seen that the tracking performance also meet the expected requirements and the tracking error converges to zero asymptotically. However, compared to previous fractional sliding mode controller, the tracking performance seems to be a little inferior. In order to see the tracking performance under different fractional orders and integer order visually, a universal standard is used to quantify tracking error by calculating root mean square error (rms error). The rms error reflects how much the measured value deviates from the true value. The smaller the rms error is, the higher the measurement accuracy is. So, it can be a criterion to assess the tracking performance of the control scheme under different orders. Table 1 shows the rms errors along x-axis and y-axis under different fractional orders.

**Table 1:** RMS errors of x and y axis under different fractional orders.

RMS ERROR *α*	*X*	*Y*
0.1	0.0134	0.0121
0.3	0.0156	0.0125
0.5	0.0157	0.0120
0.7	0.0149	0.0133
0.9	0.0129	0.0118

For fairness, the fractional order *α* is added in a ladder-type increase and the integer order is set *α* = 1. It is intuitive to see that the fractional order has impact on tracking errors. When fractional order *α* = 0.9, the rms error seems to be minimal that is why we choose *α* = 0.9 in previous design procedure. In the case of *α* = 1, the rms errors along x-axis and y-axis are 0.0154 and 0.0131 which are slightly larger than the case of fractional order *α* = 0.9. This effectively verified that the adaptive fractional fuzzy sliding mode control based on backstepping technique is superior to the conventional integer order ones.

**Remark:** The computational cost of the proposed fractional order sliding mode control and traditional integer order sliding mode control is about 2 minutes and 70 seconds.

## Conclusion

An adaptive fractional fuzzy sliding mode controller for microgyroscope system based on backstepping design is presented in this paper. The object of the controller design is to make the output trajectory of microgyroscope track the reference trajectory accurately and effectively. Compared to the earlier control methods such as AGC technique and PLL technique, the proposed technique has advantages in terms of control accuracy and adaptability in engineering applications. Unlike traditional SMC with integer order, a fractional differential sliding surface is proposed which has more design freedom. Then a fuzzy system is incorporated into fractional sliding mode control to attenuate the chattering in the sliding phase. Furthermore, adaptive estimators are used to identify the angular velocity and other unknown system parameters. In order to find the best fractional order *α* for the system, simulations under different fractional orders are carried out, verifying the efficacy of the proposed control schemes. In the future research, we will focus on the design of hardware circuits and control method, build a test platform and complete the test of the microgyroscope system based on FPGA.
